# Favourable outcome of severe, unstable grade III slipped capital femoral epiphysis managed by closed reduction percutaneous pinning with mid-term follow up: A case report and literature review

**DOI:** 10.1016/j.ijscr.2024.110264

**Published:** 2024-09-19

**Authors:** Aryadi Kurniawan, Mulki Hakam, Larasati Putri Aryandhani, Witantra Dhamar Hutami

**Affiliations:** a Consultant Pediatric Orthopaedic Surgeon, Department of Orthopaedic & Traumatology, Dr. Cipto Mangunkusumo National Central Public Hospital and Faculty of Medicine, Universitas Indonesia, Jalan Diponegoro No. 71, Jakarta Pusat, Jakarta 10430, Indonesia; b Faculty of Medicine Universitas Pembangunan Nasional “Veteran Jakarta”, Jalan Pangkal Jati, Pd. Labu, Kec. Cilandak, Kota Jakarta Selatan, Jakarta 12450, Indonesia; c Rumah Sakit Khusus Bedah Rawamangun, Jalan Balai Pustaka No. 29-31, Rawamangun, Kec. Pulo Gadung, Kota Jakarta Timur, Jakarta 13220, Indonesia; d Orthopaedic Surgeon, Department of Orthopaedic & Traumatology, Dr. Cipto Mangunkusumo National Central Public Hospital and Faculty of Medicine, Universitas Indonesia, Jalan Diponegoro No. 71, Jakarta Pusat, Jakarta 10430, Indonesia

**Keywords:** Slipped capital femoral epiphysis, Closed reduction and percutaneous pinning, Mid-term follow up

## Abstract

**Introduction and importance:**

Slipped capital femoral epiphysis (SCFE) is one of the most common hip pathology in adolescents. Outcome of SCFE management largely depends on the grading of the pathology. Severe, unstable SCFE poses high risk for avascular necrosis (AVN). The objective of this study is to report a good outcome without AVN in an acute, unstable, high grade SCFE managed by closed reduction and percutaneous pinning along with factors that need to be given consideration.

**Case presentation:**

A 13 years old boy was unable to bear weight due to severe pain on right hip after trauma since 2 weeks. Patient was diagnosed with acute, unstable high grade SCFE, patient underwent closed reduction and percutaneous pinning (CRPP) using cannulated screw and K wire augmented with spica cast. At 12 weeks patient was already fully active. At 18 month follow up there was no sign of AVN with full hip range of movement.

**Clinical discussion:**

Treatment for unstable, severe SCFE is still challenging. Unreduced severe slippage will deliver serious impingement and end up with early degenerative arthritis. The magnitude of reduction in a severe, unstable SCFE poses high risk for AVN. While some studies claimed reduction is justified only when it is serendipitous, we successfully and purposely performed CRPP.

**Conclusion:**

Even after 2 weeks from onset, an acute and severe slippage can still be reduced closely and fixed percutaneously. The hip regain full range of motion with no sign of AVN on x ray at 18 months follow up.

## Highlights

• Outcome of SCFE management depends on the grading of the pathology.
• Severe, unstable SCFE poses high risk for avascular necrosis.
• Successful treatment of severe SCFE can be achieved by closed reduction and percutaneous pinning (CRPP).
• CRPP can be performed using cannulated screw and K wire augmented with spica cast.

## 1 Introduction

Slipped capital femoral epiphysis (SCFE) is a disorder in children and adolescent which consists of posteroinferior migration of the epiphysis in metaphysis through the physis of the proximal femur [1]. Depending upon sex and ethnicity, the incidence of SCFE ranges from 0.33 to 24.58 in 100,000 children of 8–15 years of age. The relative frequency of ethnicity (Caucasian is 1.0) id 5.6 for Polynesian, 3.9 for blacks, and 2.5 for Hispanics [2,3]. Recent epidemiology study found that the incidence of SCFE in Asia was 6 cases in 100,000 children of that age group [4]. Overall, the average age is 12.0 years for boys and 11.2 years for girls, and obese children present earlier than lightweight children [1]. Boys are more affected than girls, and overall peak of presentation occurs in mid-August. This is due to as the average temperature increases, a less prominent double peak has been noticed. These seasonal variations are thought to be linked to differences in vitamin D production and levels at different times of the year. The prevalence of vitamin D insufficiency or deficiency in children and adolescents is higher in blacks and/or obese children than in Caucasian and/or nonobese ones [2,3].

The causes of SCFE are divided into two types, biomechanical and biochemical. In biomechanics, obesity is the biggest cause of SCFE [5]. Meanwhile, biochemicals are related to endocrine problems, such as hypothyroidism and hypogonadism. Mechanical factor is explained as follow: the shear load to failure increases with age, and is dependent to the cross sectional diameter of the growth plate. The height and growth plate diameter have not increased proportionally with the body weight and therefore the shear load to failure expressed as a multiple of body weight is reduced. Obesity alone can reduce the resistance of the proximal femoral growth plate by up to 20 % [6]. Also, in obese patient, insulin resistance with hyperinsulinism may have an IGF-1 mimic effect on the IGF-1 receptor, increasing the susceptibility to SCFE. Obesity also results in elevated leptin levels and there has been an association with increased width of the proliferative zone with SCFE [7,8]. Hypothyroidism is the most commonly associated endocrine abnormality related to SCFE. Hypothyroidism results in an increased risk of SCFE through the proteins that might be involved in the regulation of pubertal growth due to a reduced expression of Indian hedgehog–parathyroid hormone-related hormone causing altered regulation of chondrocyte differentiation and osteocyte maturation [9].

Slipped capital femoral epiphysis is classified based on stability of the disease and temporal or timing. Based on patient's ability to walk, SCFE is divided into stable and unstable types. A patient with stable SCFE is usually an obese teenager with a brief history of poorly localized pain in the hip, groin, thigh, or knee. History of a traumatic event is rare, and the patient may also present with a mild limp, gait with external rotation of the foot, limitation of internal rotation of the hip, or with fixed position in external rotation and flexion of the hip (Drehmann sign). A patient with unstable SCFE, in other side, often has severe hip pain that does not allow gait or weightbearing. The stability classification is important, as 20– 27 % patients with unstable SCFE developed avascular necrosis (AVN) compared to only 5 % in stable SCFE [10,11]. History is often positive for hip, thigh, and knee pain and previous trauma (of a minor entity that does not justify the condition). When the patient is examined in supine position, patient shows attitude in external rotation of the side affected and counteracts any passive movement of the hip with obligatory external rotation of the hip is noted when it is passively flexed to 90° [1]. SCFE can also be divided into acute, acute-on-chronic, and chronic. Acute SCFE is characterized by sudden epiphyseal displacement and the presence of symptoms for <3 weeks. Chronic SCFE is characterized by symptoms presenting for >3 weeks, with remission and relapse. Acute-on-chronic SCFE is diagnosed when symptoms occur abruptly with exacerbation of pain and inability to walk, with lower-limb pain for >3 weeks. According to degree of slippage, the Wilson method measures the relative displacement of the epiphysis on the metaphysis in a frog-leg lateral radiograph. A grade I slip involves epiphysis displacement less than one third of the width of the metaphysis. A grade II slip involves displacement between one third and one half of the width and a grade III severe slip involves displacement greater than one half of the width [12].

Once SCFE is diagnosed, surgical treatment is indicated, but there are still controversies regarding the best treatment. The goals of treatment are to prevent further slippage and correct the deformity, thus avoiding osteonecrosis and chondrolysis. The surgical choices for SCFE can be in situ fixation with or without prophylactic fixation in selected cases, osteotomy, closed reduction and percutaneous pinning (CRPP), or surgical hip dislocation. Literature mentioned that in situ fixation is the gold standard treatment, however, emergent closed or open reduction with fixation is advocated to mitigate the risk of developing early arthritis [13]. The outcome largely depends on appropriate management which based on the grading of the pathology. Some SCFE cases are insidious rendering severe and late stage before getting medical attention. Such late stage makes surgical management poses high risk of complications.

While the established treatment for stable and low grade SCFE is in situ pinning, the treatment for unstable and severe, high grade SCFE is still in controversy. The nature of instability obviously warrants stabilization. In situ pinning in severe, high grade SCFE will end up in femoroacetabular impingement that eventually deliver early degenerative arthritis. Modified Dunn procedure offers opportunity to perform reduction and internal fixation with acceptable outcome even though there are still cases with AVN post operatively. Kohno et al [14] and Veramuthu et al [15] stated that the rate of AVN in unstable SCFE treated by closed reduction and pinning was 21,9–35 % (respectively) compared to around 18,5 % in open reduction and pinning.

Our case presented a severe, unstable SCFE. The characteristic of this kind of case is on how to choose the procedure and how the procedure must be performed in order to avoid complication. The objective of this study is to report a favourable outcome of severe unstable high grade SCFE managed by closed reduction and percutaneous pinning and factors which require special attention and consideration. The methodology of this case report had been in line with SCARE criteria [16].

## 2 Patient history

A 13 years old boy was brought to the clinic with inability to bear weight due to severe pain on right hip. Two weeks previously, patient was getting into a skid from stair at school (visual analog scale – VAS of 7/10). Based on history, patient was jumping on the stair and then he felt in sitting position. After the fell patient was difficult to bear weight with pain on from the hip down to the knee. Twelve hours before clinic visit, he fell again while walking, with inability to walk and deformity on the affected extremity. Patient had no history of allergy and had no family history of the same complaints.

## 3 Clinical finding

On arrival, the patient was alert and well-oriented. He complained of an inability to stand and severe pain in the right hip which radiated down into the knee. Physical examination revealed an externally rotated and shortened right lower limb with limitation in the range of movement. The body mass index of the patient was 23.6, indicating that the patient was overweight.

## 4 Timeline

**Table:** 

Time	Clinical finding	Treatment
Two weeks before hospital admission	Pain on the right hip after a patient fell into a sitting position on the stairs while jumping. Pain from the hip radiated down to the knee, with difficulty to bear weight.	His family brought him to a traditional massage.
4 days after initial trauma	The pain was not subsided, and then the patient was brought to a local hospital. Conclusion at that time no abnormalities were detected on x-ray (Fig. 1). The clinical diagnosis was soft tissue contusion.	The patient was given analgetic and discharged home.
12 h before hospital admission	Pain on the right hip with inability to bear weight after falling while walking. Externally rotated and shortened right lower limb. The radiograph showed severe slip of the right capital epiphysis	Planned to have an urgent closed reduction and percutaneous pinning with possibility of Modified Dunn procedure if closed reduction failed
2 months after surgery	No pain on the right hip, normal range of motion. Radiograph showed maintenance of the reduction and fixation	The patient was allowed to fully bear weight
5 months after surgery	No pain on the right hip, normal range of motion. Radiograph showed maintenance of the reduction, with a broken k-wire due to increased activity	K-wire removal
18 months after surgery	No pain on the right hip, normal range of motion. Radiograph showed maintenance of the reduction, with no sign of AVN	The patient was allowed to have full activity

## 5 Diagnostic assessment

Anteroposterior (AP) pelvic radiograph after the second trauma is shown in Fig. 2. Based on the clinical and radiograph findings after the second trauma, the patient was diagnosed with acute, unstable grade III SCFE. In the AP pelvic radiograph after the initial trauma, there was decreased epiphyseal height on the right hip compared to the left hip. Also on the right hip, Klein's line did not cross the epiphysis. In the AP pelvic radiograph after the second trauma, there was clear posteroinferior migration of the epiphysis in metaphysis through the physis of the proximal femur on the right hip with widening of the growth plate (physis) compared to the left hip. On the right hip also showed that the Klein's line did not cross the epiphysis. The measurement of posterior sloping angle (PSA) showed value of 25°. Thyroid panel was examined on the patient and the result showed normal value.

**Fig. 1:**
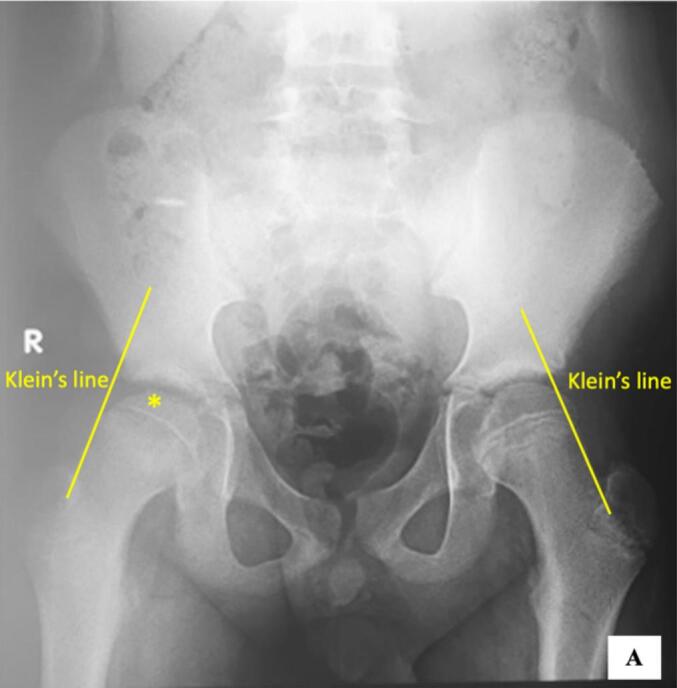
First anteroposterior pelvic plain radiograph. Depicted the AP pelvic radiograph after the first trauma. In the image, the asterix sign showed decreased epiphyseal height on the right hip compared to the left hip. Also on the right hip, Klein’s line did not cross the epiphysis.

**Fig. 2:**
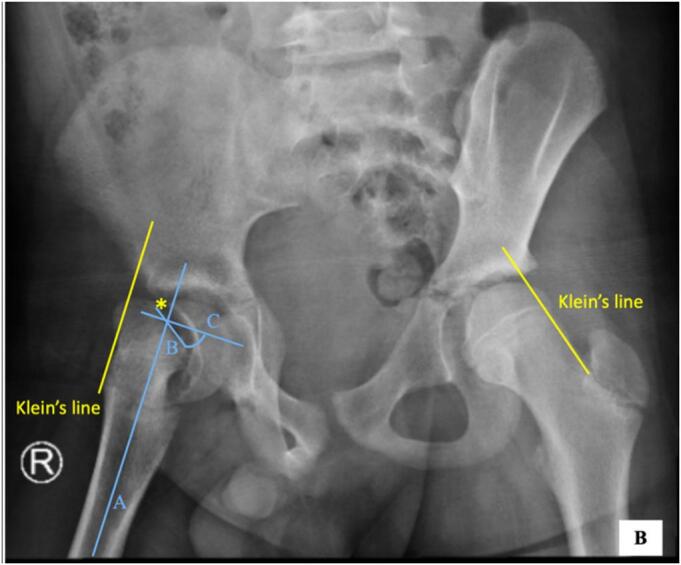
Anteroposterior pelvic X ray after the second trauma. In the image, there was clear posteroinferior migration of the epiphysis in metaphysis through the physis of the proximal femur on the right hip. Asterix sign showed widening of the growth plate (physis) compared to the left hip. On the right hip also showed that the Klein’s line did not cross the epiphysis. The measurement of posterior sloping angle (PSA)showed line A as a line along femoral neck-diaphyseal axis, line B as plane of physis, and line C as a line perpendicular to line A. PSA is measured as angle subtended between B and C, this measured as 25 degree.

**Fig. 3:**
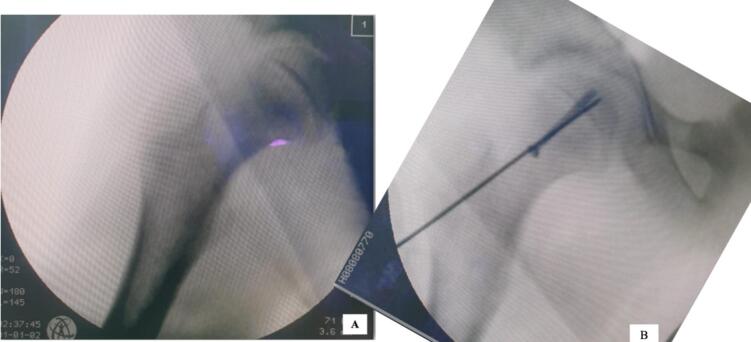
Closed reduction and percutaneous pinning under image intensifier. (A) patient was positioned supine in traction table. Closed reduction was first performed by applying longitudinal traction and internal rotation of the right lower extremity, until satisfying reduction was achieved as seen on intraoperative image intensifier. (B) entry point was confirmed using a 2.0 mm K-wire, the trajectory of the K-wire was anteromedially to allow for maximum engagement to the femoral head. The skin and facia were incised, and a meticulous subfascial dissection was performed to gain access to the anterior femur. A cannulated guide wire was inserted, its position was confirmed by image intensifier. The guide wire was placed in the centre of the head and measured, and a 7.3 mm partially threaded screw was inserted. A k-wire was also inserted just superior and parallel to the first to achieve greater biomechanical stability.

**Fig. 4:**
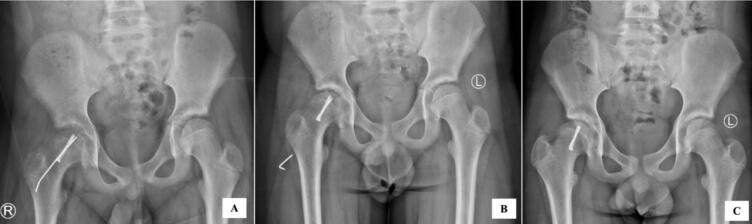
Radiographic images during follow up. (A) in 2 months after surgery, radiograph showed maintenance of the position of the right proximal femoral physis with pain free and full range of motion of the right hip, thus patient was allowed to fully bear weight. (B) at the follow up of 5 months after surgery, radiograph showed broken k-wire, however the reduction was maintained. Patient had been free of pain and had full hip range of motion. (C) at the final follow up of 18 months after surgery, radiograph showed maintenance of the reduction with healing of the right proximal femoral physis and no sign of AVN. Patient had been free of pain and had full hip range of motion, thus patient was allowed to have full activity.

**Fig. 5:**
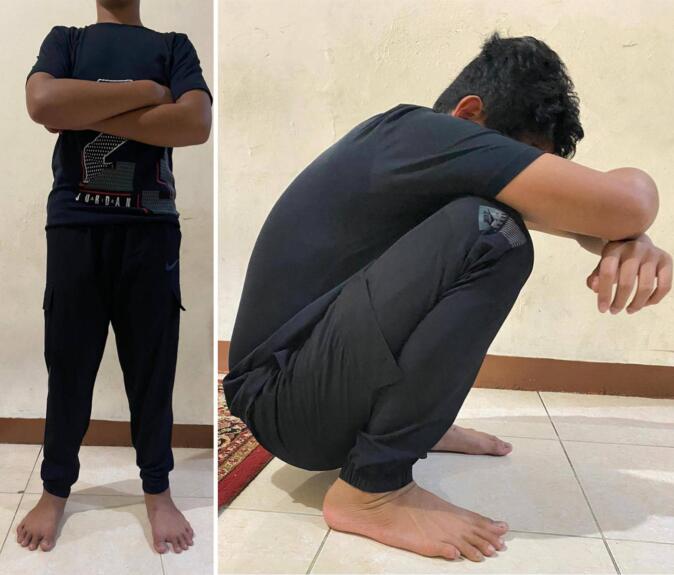
Clinical appearance of the patient at final follow up. At final follow up, patient is able to stand, walk, run, and squat without symptoms.

## 6 Therapeutic intervention

Emergent closed reduction and percutaneous pinning were performed under an image intensifier. The surgical procedure was as follow: the patient was positioned supine in traction table. Closed reduction was first performed by applying longitudinal traction and internal rotation of the right lower extremity until satisfying reduction was achieved as seen on intraoperative image intensifier. This closed reduction was performed gently and purposefully in one attempt. Surgery was proceeded with fixation using percutaneous pinning under image intensifier guidance. The technique is described in detail as follow:

1. Guidewire placement

The goal of guidewire placement is to place the tip of the wire perpendicular to the physis in the middle third of the epiphysis, that is about 3 mm from subchondral bone. The starting point is on the anterior neck, in order to properly place the wire in the middle of the epiphysis. Guide wire is placed on the anterior hip, and the antero-posterior (AP) image intensifier is used to make sure that the wire is aligned over the center of the femoral head. Then, skin is marked at the tip to identify the position of the femoral head center, and the line is followed laterally to the lateral aspect of the femur. This line represents the course along which the guide pin for the bone screw will follow.

2. Skin incision

A 1-cm skin incision is made through the skin and spread with a hemostat down to bone along the drawn line but at an angle of 30° toward the horizontal (the head-neck angle). The skin and facia were incised, and a meticulous subfascial dissection was performed to gain access to the anterior femur.

3. Guidewire insertion

The guidewire is inserted into the femoral neck, then advanced to within 3 mm of the articular surface, continued with measurement of the wire to determine the screw length

4. Screw insertion

The cannulated drill is drilled over the guidewire. The drill is stopped 1 or 2 mm before the tip of the guidewire to keep the guidewire in place. The bone is tapped with the cannulated tap over the guidewire. The 6.5- to 7.5-mm (we used 7.3 mm screw) cannulated screw, partially threaded is placed over the guidewire. The guidewire is then removed. A k-wire was also inserted just superior and parallel to the first to achieve greater biomechanical stability (Fig. 3). The lower extremity was then abducted and exorotated to confirm stability.

Augmentation using hip spica cast was applied for 3 weeks. Postoperatively, patient was told not to bear weight for 6 weeks.

## 7 Follow-up and outcomes

The patient was followed up at 2nd, 5th, and 18th months after surgery. Initially, patient was refrained to bear weight for 6 weeks, thereafter patient was allowed to partially weight bear. In 2 months after surgery, the radiograph showed maintenance of the position of the right proximal femoral physis with pain free and full range of motion of the right hip, thus patient was allowed to fully bear weight (Fig. 4). At the follow up of 5 months after surgery, radiograph showed broken k-wire, however the reduction was maintained. Patient had been free of pain and had full hip range of motion, thus patient initiatively played football. Due to broken k-wire, surgery for removal of the k-wire was performed. At the final follow up of 18 months after surgery, radiograph showed maintenance of the reduction with healing of the right proximal femoral physis and no sign of avascular necrosis (AVN). Patient had been free of pain and had full hip range of motion, thus patient was allowed to have full activity. Clinically at final follow up, patient was able to participate in full activity, with no complaints during standing and squatting (Fig. 5).

## 8 Discussion

Slipped capital femoral epiphysis (SCFE) is a well-recognized disorder affecting adolescents which has potential long-term sequelae that may permanently alter hip function [17]. Patients with acute SCFE is considered at increased risk for developing AVN of the upper femoral epiphysis, thus the choice of technique and method used to stabilize or reposition the epiphysis affected the function and survivorship of the treated hip [18]. Avascular necrosis is the most serious complication of SCFE. Kohno et al [14] performed a research study which concluded that 27 % of unstable SCFE patients that treated by closed reduction and pinning than those treated by in situ pinning. Another systematic review by Veramuthu et al [15] showed that the risk of developing AVN following closed pinning compared to open reduction in patients with unstable SCFE was not significant. The prevalence of osteonecrosis in unstable SCFE patients following closed pinning and open reduction were 21.9 % and 18.5 %. The risk ratio for AVN with intentional closed reduction was 1.21 times higher than with the modified Dunn method, even though no significant difference was noted.

Other complications that can occur following SCFE are impingement, chondrolysis and slip progression. A SCFE is considered as stable if the patient is able to walk on the affected limb, either with or without walking aids. Unstable SCFE is when the patient is not able to walk on the affected limb, even with the help of aids, regardless the duration of symptoms. The distinction between stable and unstable SCFE is important regarding the prognosis and choice of treatment. Other than based on stability, onset of symptom, and slip grade, SCFE is also classified based on Southwick slip angle, a femoral head-neck angle measured in the correct frog-leg lateral position [19]. This angle is measured as the angle between the neck and head axes on a frog-leg lateral radiograph, which is determined by subtracting the angle between the head axis and the femoral shaft axis in the unaffected side from the affected side.

Once diagnosed, the treatment of SCFE aims to stop the slip progression, relieving impingement, and preventing or delaying OA, and importantly, preventing AVN. According to a systematic review study, the best method of treatment for the stable SCFE is in situ pinning with a single central screw, this procedure was associated with the lowest incidence of complications [20]. Mahran et al [18] proposed a treatment algorithm for patient with SCFE. In stable SCFE with slip angle of less than 30° with no impingement, in situ fixation is the choice of treatment. If impingement is present in SCFE with slip angle of less than 30°, in situ fixation with osteochondroplasty by mini-open, arthroscopic, or surgical hip dislocation technique (SHD). In stable SCFE with slip angle of 30° or more and open physis, the choice of treatment is SHD and modified Dunn procedure (subcapital realignment). Stable SCFE with slip angle of 30° or more and closed physis, combined intertrochanteric osteotomy (ITO) and osteochondroplasty is the treatment choice. In unstable SCFE, modified Dunn procedure through SHD is indicated.

Gentle closed reduction is a simple treatment option in unstable SCFE with risk of AVN comparable to that of other treatments. While this procedure is much less invasive and technically challenging than other procedures, it can be considered a reasonable alternative to SHD. Napora et al [21] in their retrospective cohort concluded that stable SCFEs are treated with in situ pinning, while unstable SCFEs can be treated with a gentle but purposeful closed reduction technique. The patient is placed supine on a fracture table ad application of longitudinal traction, with the affected leg is mildly internally rotated with the goal of the patella achieving an anterior position toward the ceiling and generally not passing this point to allow for a gentle reduction, then, in situ pinning is performed with one or two screws. The important thing that should be highlighted here is the gentle and purposeful, in contrary to inadvertent closed reduction, should be performed to avoid the risk of AVN [22].

We presented a case with unstable, high grade SCFE and treated the patient with closed reduction and percutaneous pinning (CRPP), with favourable outcomes. According to clinical and radiological data, we suggested that initially, patient suffered from acute, stable SCFE which was missed. This is based on the risk factors of SCFE, the obesity and active child, presented in the patient and the initial plain radiograph showed Klein's line which did not pass the capital femoral physis. Due to continued weight bearing and no intervention, this stable SCFE progressed into unstable, high slip grade SCFE with slip angle of more than 30°. This unstable type of SCFE is in line with the clinical manifestation of inability to bear weight. If the patient came with initial condition, in situ pinning is planned to the patient. As the untreated disease progressed, a modified Dunn procedure through SHD is indicated, as mentioned on the treatment algorithm. However, the literature mentioned that for unstable SCFE, the surgical treatment is still controversial as many factors influenced the decision [18]. Beside modified Dunn procedure through SHD, CRPP also can be indicated in patient with acute, unstable SCFE. In 2018, Novais et al [23] performed a study to compare modified Dunn procedure versus CRPP in patients with unstable SCFE. They found that both procedures showed good or excellent outcomes, with no significant difference between those two groups. Also, they found that incidence of AVN and unplanned re-operation did not differ significantly between modified Dunn procedure versus CRPP, despite in modified Dunn procedure showing better improvement in Southwick slip angle. A survey study found that the preferred method for the treatment of unstable SCFE among members of the Paediatric Orthopaedic Society of North America (POSNA) is percutaneous fixation after positioning the hip to neutral rotation [24]. Furthermore, Cheok et al [13] in 2023 presented a rare, acute unstable valgus slip of SCFE. They treated the patient with CRPP with satisfactory outcomes in 18 months after surgery. Our case showed that after performing CRPP to acute, unstable high grade SCFE, patient had favourable clinical outcomes with no sign of AVN, which is satisfactory.

The next question is did the patient need prophylactic pinning for a contralateral, healthy hip? The contralateral hip in otherwise healthy patients presenting with a unilateral symptomatic SCFE remains a controversial subject. Some studies have identified the risk factors of developing sequential contralateral involvement in unilateral SCFE. The most significant factors that were associated with a contralateral slip were BMI greater than the 95th percentile and a younger age at presentation [25]. Increases in PSA (higher than 16.5°) have been proposed to be predictive of contralateral slip in SCFE. The PSA of the physis is defined as the angle between the line along the plane of the physis and the line perpendicular to the femoral neck- diaphyseal axis on an axial radiograph [26]. Regarding the question of prophylactic pinning for contralateral, healthy hip, there are two choices available: to perform prophylactic pinning or to perform a ‘wait and see’ approach [27]. The advantages of prophylactic pinning of the unaffected side in otherwise healthy patients with unilateral SCFE outweigh the disadvantages. However, the final decision for treatment remains to be patient-specific [28]. For the conclusion, prophylactic pinning is indicated in patients with risk factors of obesity, high affected PSA before slipping, and endocrinopathy. Careful observation until growth plate closure is required in patients without risk factors [29]. As can be seen in our case, the patient presented with obesity and high PSA. However, the laboratory endocrine marker showed normal value, thus ‘wait and see’ approach was chosen for this patient. At 18 months after surgery, no contralateral slip occurred in the patient. In this patient, broken k-wire remaining on the femoral head was not removed. Routine implant removal is controversial, however, attempt to remove implant is to reduce risk of fracture associated with an increased stress riser, screw irritation, or in preparation for future hip procedures [30]. Due to broken k-wire, we removed only to the part of the k-wire positioned on the soft tissue.

Several matters need to be considered in determining treatment for patient with SCFE. Closed reduction is contraindicated for stable SCFE, due to continuity between the epiphysis and metaphysis has not been disrupted. Attempted reduction may fail and there is the risk of inadvertent iatrogenic physeal destabilization. In unstable SCFE, the epiphysis separates from the metaphysis, causing the vessels supplying the epiphysis to rupture, kink, and stretch. This eventually places the femoral head to be in high in risk of AVN [31]. Second, the closed reduction must be performed gently and purposefully in order to prevent the risk of AVN, and if the purposefully reduction fail after first attempt, the SHD must be performed thereafter.

## 9 Conclusion

Even after 2 weeks from onset, an acute and severe slippage can still be reduced closely and fixed percutaneously. The hip regained full range of motion with no sign of AVN and contralateral slip on x-ray at 18 months follow up. This report showed that closed reduction and percutaneous pinning can be successfully applied in acute, unstable high grade SCFE. This favourable outcome comes under condition of proper planning of reduction, gentle when doing it, single attempt, and proper placement of the implant.

## Patient perspective

Postoperatively, patient and his parents are satisfied with the treatment outcomes.

## Informed consent

Written informed consent was obtained from the patient's parents for publication and any accompanying images. A copy of the written consent is available for review by the Editor-in-Chief of this journal on request.

## Ethical approval

Ethics approval is not required for this case reports deemed not to constitute research at the institution. The institution is Faculty of Medicine Universitas Indonesia – Dr. Cipto Mangunkusumo National Central Public Hospital.

## Funding

The authors received no financial support for the research, authorship, and/or publication of this article.

## Author contribution

Aryadi Kurniawan: study concept, data collection, data interpretation, and writing the paper.

Mulki Hakam: data collection, data interpretation and writing the paper

Larasati P Aryandhani: data collection, data interpretation and writing the paper

Witantra Dhamar Hutami: data collection, data interpretation and writing the paper.

## Guarantor

Aryadi Kurniawan.

## Research registration number

This case report is not a first in man study.

## Conflict of interest statement

The authors certify that They have NO affiliations with or involvement in any organization or entity with any financial interest or non-financial interest in the subject matter or materials discussed in this manuscript.
